# iNOS Activation Regulates β-catenin Association with Its Partners in Endothelial Cells

**DOI:** 10.1371/journal.pone.0052964

**Published:** 2012-12-28

**Authors:** Deyarina Gonzalez, Armando Rojas, Maria Beatriz Herrera, R. Steven Conlan

**Affiliations:** 1 Centre for NanoHealth, College of Medicine, Swansea University, Swansea, United Kingdom; 2 Biomedical Research Lab, School of Medicine, Catholic University of Maule, Talca, Chile; 3 Department of Internal Medicine, Research Centre for Experimental Medicine, University of Torino, Torino, Italy; Biological Research Centre of the Hungarian Academy of Sciences, Hungary

## Abstract

**Background:**

Signals that disrupt β-catenin association to cadherins may influence the translocation of β-catenin to the nucleus to regulate transcription. Post-translational modification of proteins is a signalling event that may lead to changes in structural conformation, association or function of the target proteins. NO and its derivatives induce nitration of proteins during inflammation. It has been described that animals treated with NO donors showed increased permeability due to modulation of VE-cadherin/catenin complex. We, therefore, aim to evaluate the effect of iNOS activation on the expression, nuclear localisation and function of β-catenin in endothelial cells.

**Methodology/Principal Findings:**

Expression, nuclear localisation, post-translational modifications and function of β-catenin was analysed by cell fractionation, immunoprecipitation, immunoblots, QRT-PCR and permeability assays in murine endothelial cells (H5V). Influence of macrophage activation on expression of VE-cadherin/p120-catenin/β-catenin complex in co-cultured H5V cells was also assessed. Activation of macrophages to produce NO provoked a decrease in VE-cadherin/p120-catenin/β-catenin expression in H5V cells. Phosphorylation of β-catenin, p120-catenin and VE-cadherin, and reduction in the barrier properties of the cell monolayer was associated with iNOS induction. Moreover, high NO levels provoked nitration of β-catenin, and induced its translocation to the nucleus. In the nucleus of NOS activated cells, nitration levels of β-catenin influenced its association with TCF4 and p65 proteins. High levels of NO altered β-catenin mediated gene expression of NFκB and Wnt target genes without affecting cell viability.

**Conclusions:**

NOS activity modulates β-catenin post-translational modifications, function and its association with different partners to promote endothelial cell survival. Therapeutic manipulation of iNOS levels may remove a critical cytoprotective mechanism of importance in tumour angiogenesis.

## Introduction

Nitric oxide (NO), a free radical that mediates cytotoxic effects against host tissues and cells, plays a vital role in the regulation of inflammation. Detrimental effects of NO that are observed in the advanced stages of the inflammatory process include tissue injury and exacerbation of inflammation through activation of inducible nitric oxide synthases (iNOS) [1], [2]. Chronic inflammatory diseases such as diabetes, arthritis, ulcerative colitis, Crohn’s disease, septic shock, and atherosclerosis are associated with excessive production of NO and its derivatives [2], [3]. NO exerts many of its functions through post-translational modification of proteins, affecting signalling pathways by modifying protein-protein interactions [4], [5]. Protein tyrosine phosphorylation and nitration are among the NO-mediated protein modifications that accompany inflammatory processes [6]. In this context, β-catenin is emerging as a key target for NO actions. Nonsteroidal anti-inflammatory drugs, like NO donating aspirin (NO-ASA), promote S-nitrosylation of β-catenin as well as tyrosine nitration of proteins expressed in human colon cell lines [7]. In endothelial and epithelial cells, incubations with peroxynitrite, a NO derivative, or the NO donor glycerol trinitrate (GTN), promote nitration of β-catenin leading to increases in vascular permeability or altered β-catenin transcriptional activity [8], [9].

β-catenin is a ubiquitously expressed protein that plays at least two important functions in the cell. First, as a protein located at cell-cell adherent junctions (AJ) associated with cadherins (VE- and N-cadherin in endothelial cells) stabilizing their association with the cytoskeleton [10]. Second, as a transcriptional activator of the Wnt signalling pathway, associated with T-cell factor (TCF)/Lef transcription factors; governing cell proliferation, differentiation, survival and fate [11]. Other nuclear partners of β-catenin include the transcriptional factor NFκB, which participates in the induction of genes involved in immunity, apoptosis and inflammation including iNOS [12], [13].

In the cytoplasm, β-catenin associates with a destruction complex composed of tumour suppressor protein adenomatous polyposis coli (APC), axin proteins, and serine-threonine glycogen synthase kinase-3β (GSK-3β) [11]. This destruction complex phosphorylates β-catenin and targets it for degradation by means of the proteasome. Wnt signalling inhibits the destruction complex and promotes β-catenin nuclear translocation [11]. Cadherins can promote β-catenin binding to the complex, inhibiting Wnt signalling [14]. Cadherins can limit β-catenin signalling by modulating its nuclear availability. Tyrosine phosphorylation of VE-cadherin, p120-catenin or β-catenin also promotes AJ dissociation, increases in vascular permeability and could potentially influence the translocation of β-catenin to the nucleus [15]. It has been reported that inflammatory mediators, including thrombin and vascular endothelial growth factor (VEGF), stimulate tyrosine phosphorylation of AJs and β-catenin redistribution from AJs to the nucleus, with concomitant increases in vascular permeability without affecting cellular viability [16], [17]. It has also been shown that S-nitrosylation of β-catenin disrupts its association with TCF4, inhibiting expression of Wnt targets and promoting NO-mediated cytotoxicity [18], [19] Thus, posttranslational modification of β-catenin may strongly influence β-catenin function and cell fate.

Given that the inflammatory process is characterized by high levels of NO, a leaky endothelium and expression of NFκB pro-inflammatory target genes, we hypothesized that NO may influence β-catenin function during chronic and acute inflammatory processes. We have previously reported that NO released by NO donors modulates the expression of VE-cadherin/catenin complex and increases endothelial cell permeability both *in vitro* and *in vivo* [20]. To confirm our hypothesis, we investigated the contribution of iNOS activation on the post-translational modifications, nuclear localisation, function and association of β-catenin with its partners in endothelial cells. In this work, we also studied the expression levels and post-translational modifications of VE-cadherin and p120-catenin, partners of β-catenin that sequester it at the cell-cell junctions.

## Results

### Macrophage iNOS-derived NO Modulates the Expression of β-catenin and its Membrane Partners VE-Cadherin and p120-catenin in H5V Cells

**Figure 1:**
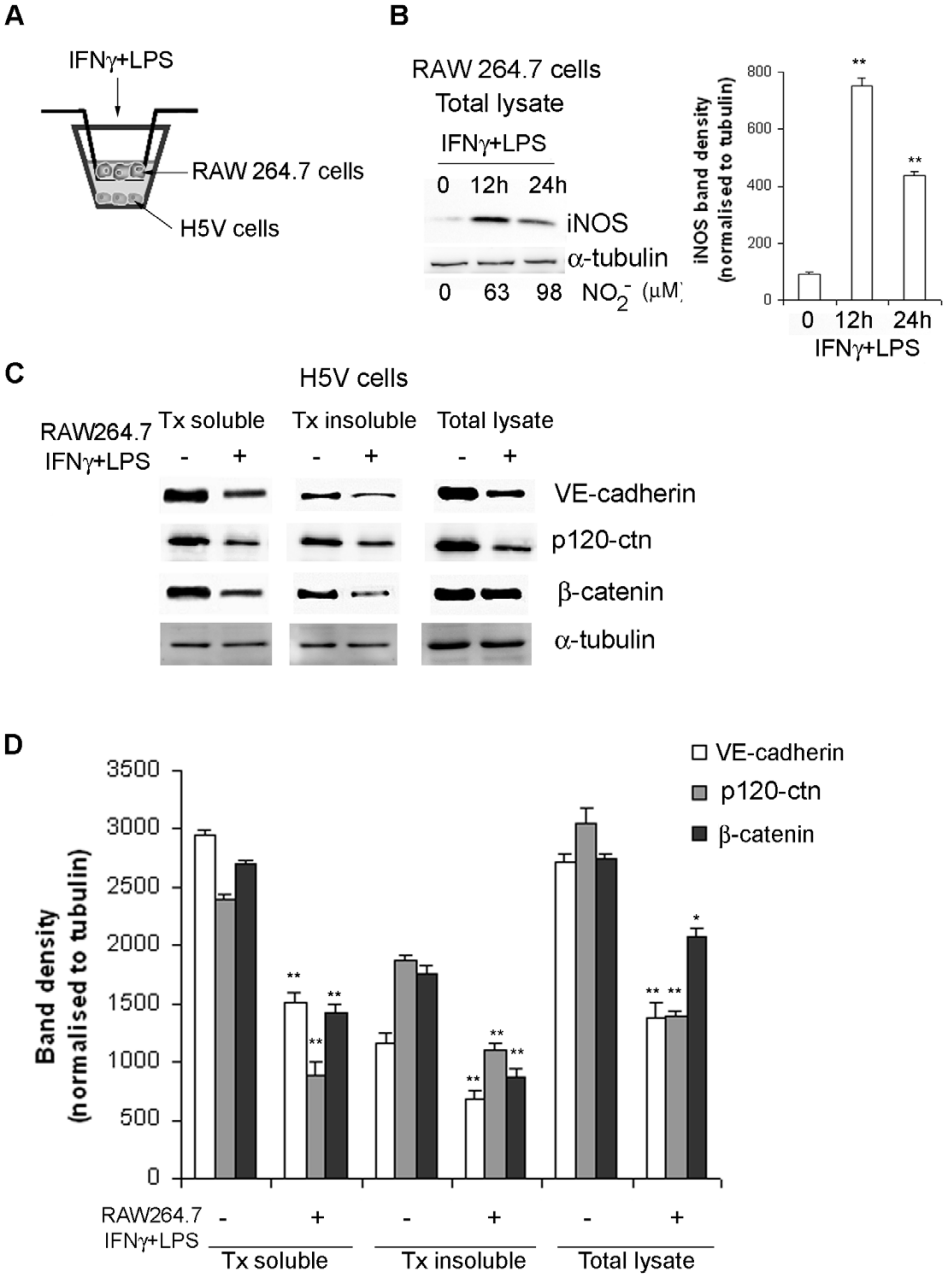
Effect of macrophage activation on VE-cadherin, p-120 catenin and β-catenin expression in H5V cells. **A.** RAW264.7 and H5V cells were grown to confluence on the upper and lower compartment of Transwell units respectively. Macrophages were stimulated with IFNγ/LPS for 12 h to produce NO and nitrite production was monitored in the media. Total cell lysates were obtained from RAW 264.7 cells grown in the upper compartment. TX-100 (TX) protein fractions, as well as total cell lysates, were obtained from H5V cells grown on the lower compartment. **B.** Induction of iNOS protein on IFNγ/LPS stimulated macrophages was determined by immunoblot with a murine iNOS mAb. Nitrite production was measured using the Griess method and nitrite concentrations expressed in µM. Bands of 130 kDa corresponding to iNOS were detected in the samples. α-tubulin levels were used as a loading control. The graph represents the western blot quantification (*P<0.05; **P<0.01). **C.** Expression of VE-cadherin, p120-catenin (p120-ctn) and β-catenin is reduced in H5V cell co-cultured (12 h) with activated macrophages. H5V Total cell lysates and TX- fractions were analysed by western blot for VE-cadherin, p120-ctn and β-catenin expression by using specific antibodies. Expression of α-tubulin was determined as a loading control. **D.** Graph represents the densitometry analysis of Panel C western blots. α-tubulin was used as a loading control. The significance level was set at P<0.05 (*P<0.05; **P<0.01).

Activated macrophages are a significant source of iNOS-derived NO at the site of inflammation [21]. Pro-inflammatory cytokines such as interferon gamma (IFNγ), and bacterial cell components (LPS) are able to trigger the expression of iNOS in macrophages and other cell types including endothelial cells (EC) [22], [23]. To address the question of whether the levels of VE-cadherin/p120-catenin/β-catenin complex might be regulated by high levels of NO in a whole-cell physiological context, RAW 264.7 macrophages and murine microvascular H5V ECs were co-cultured to confluence in the upper and lower compartments respectively of Transwell units (Fig. 1A).

Induction of iNOS expression and activity in macrophages, stimulated with the IFNγ/LPS combination, was confirmed respectively by immublots and the increase of nitrite levels in the media (Fig. 1B). In parallel we observed a statistically significant reduction on VE-cadherin, p120 catenin and β-catenin levels in both the TX-soluble (49%, 73% and 45% reduction respectively) and TX-insoluble (41%, 42% and 51% reduction respectively) fractions from H5V, co-cultured for 12 h with the IFNγ/LPS activated macrophages (Fig. 1C, D) These results are in accordance with our previous observations for NO donors in H5V cells [20]. Total levels of VE-cadherin and p120-catenin were reduced by 50% and 54% respectively whereas a 28% reduction of β-catenin levels was observed in H5V cells co-cultured for 12 h with IFNγ/LPS activated macrophages (Fig. 1C and D). As previously described for the NO donors, PECAM-1 (CD31), which co-localizes to the lateral endothelial cell junctions, remained at constant levels after NO stimulation (data not shown) [20].

### Endothelial iNOS-derived NO Modulates Expression of AJ Proteins and Barrier Properties of the Endothelium

**Figure 2:**
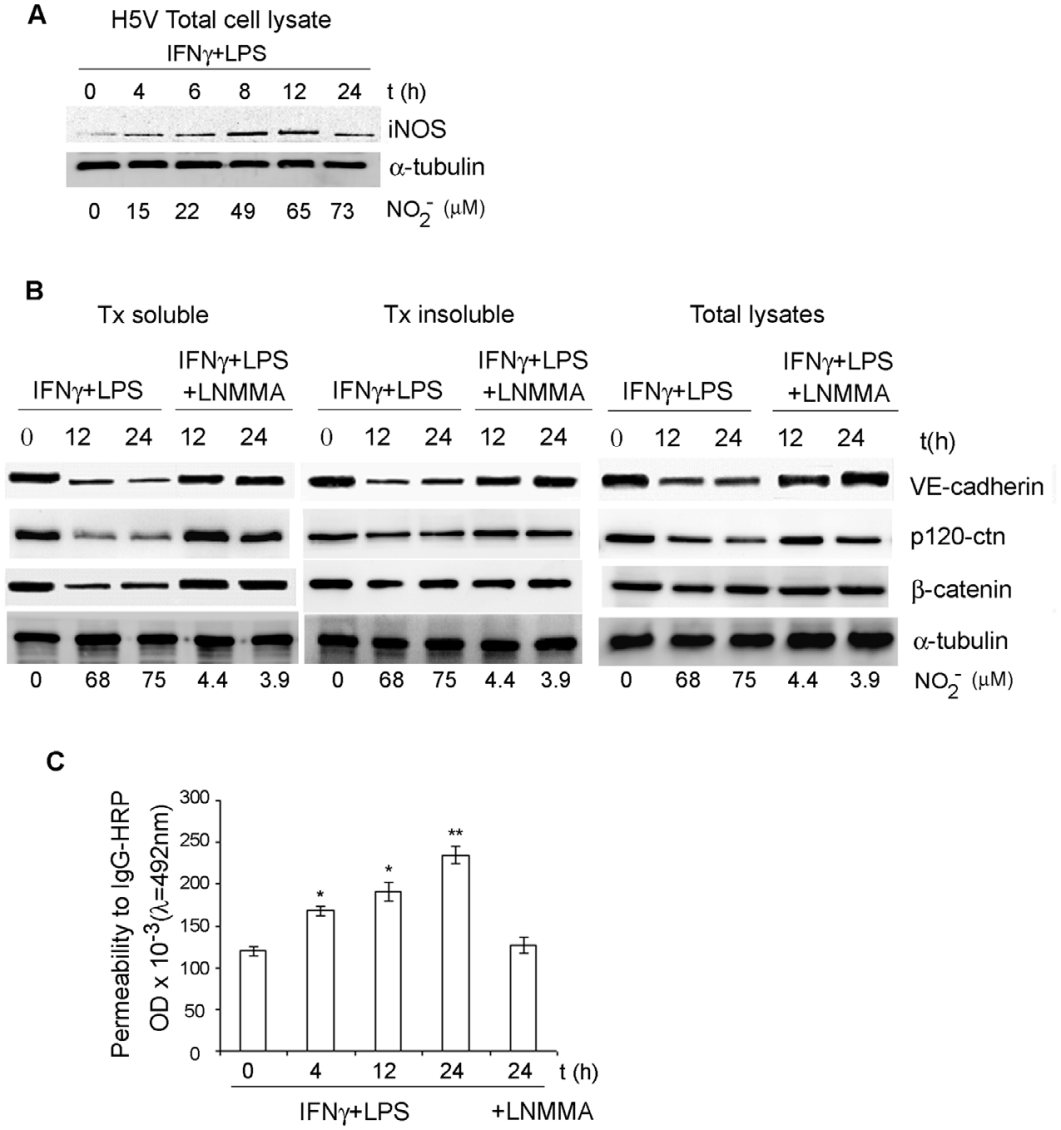
iNOS activity regulates the expression and function of VE-cadherin/p120catenin/β-catenin complex in H5V cells. **A.** Time course induction of iNOS protein on IFNγ/LPS stimulated H5V cells. Nitrite production was measured using the Griess method and nitrite concentrations expressed in µM. Bands of 130 kDa corresponding to iNOS were detected in the samples. Western blot quantification is shown in Fig. S1. **B.** Expression of VE-cadherin, p120-catenin (p120-ctn) and β-catenin is reduced in H5V cells incubated with IFNγ/LPS. NOS inhibitor LNMMA attenuates NO effect on VE-cadherin/catenin complex. H5V TX fractions and total cell lysates were analysed by western blot for VE-cadherin, p120-catenin (p120-cnt) and β-catenin levels using specific antibodies.α -tubulin levels were used as a loading control. Nitrite production was measured using the Griess method and nitrite concentrations expressed in µM. Western blot quantifications are shown in Fig. S1 (*P<0.05; **P<0.01). **C.** NO stimulates paracellular permeability to IgG-HRP in H5V cells. H5V cells were grown to confluence in Transwell units and stimulated to produce NO. Paracellular permeability of endothelial cell monolayers to IgG-HRP (200 kDa) was measured as described in methods. Control cells were incubated with LNMMA, to inhibit iNOS activation. Statistical analysis was done using a t-test. The significance level was set at P<0.05 (*P<0.05; **P<0.01).

Based on the observations described we decided to assess the effect of iNOS induction in our endothelial model. iNOS induction has been observed in endothelial cells from different vascular origins [24], [25]. To assess for iNOS induction in our experimental conditions, H5V confluent monolayers were directly treated with IFNγ/LPS for 24 h. Induction of iNOS expression was detected after 4 h of IFNγ/LPS stimulation, and decreased after 24 h exposure to IFNγ/LPS (Fig. 2A and Fig. S1). Nitrite concentration rose linearly in the culture media demonstrating activation of iNOS (Fig. 2A).

NO-dependent regulation of the VE-cadherin/p120-catenin/β-catenin complex was observed in both TX-soluble and insoluble fractions, as well as in total cell lysates (Fig. 2B and Fig. S1 for densitometry data). Activation of iNOS correlated with the dramatic reduction of VE-cadherin and p120-catenin levels in IFNγ/LPS- stimulated H5V cells (Fig. 2B). Using quantitative densitometry, NO reduced VE-cadherin levels by 84% and 71% in the TX soluble samples, by 61% and 52% in the TX insoluble samples and 53% and 48% in the total cell lysates samples (12 h and 24 h IFNγ/LPS stimulations respectively) (Fig. S1). We observed similar reductions in p120-catenin levels in TX soluble (64% and 62%), TX insoluble (63% and 61%) and total lysates (65% and 62%) samples after 12 h and 24 h IFNγ/LPS stimulations (Fig. S1). iNOS activation also reduced β-catenin levels in TX soluble (48% and 44%), TX insoluble (26% and 17%) and total lysates (20% and 18%) samples (12 h and 24 h IFNγ/LPS stimulations respectively).

The L-arginine analogue LNMMA was used to cause irreversible inactivation of the NOS enzyme [26]. Accordingly, a decrease in the levels of nitrite, that indicates the inhibition of iNOS activity, was observed after co-incubations with LNMMA. Inhibition of iNOS activity partially restored the levels of p120-catenin (7.3%, 6.8% and 7.2% reduction in TX soluble, insoluble and total lysates respectively after 24 h incubations) and VE-cadherin (20%, 16% and 18% reduction in TX- soluble, -insoluble and total lysates respectively after 12 h incubations). Incubations with LNMMA abolished NO effect on β-catenin levels (Fig. 2B and Fig. S1).

We then tested if iNOS induction provoked any alterations on the function of VE-cadherin/p120-catenin/β-catenin complex regulating barrier properties of the endothelial monolayer. Indeed, iNOS induction results in a time dependent increase in the permeability of the H5V monolayers to large biomolecules (Ig-HRP), with highest permeability values observed after 24 h iNOS activation (Fig. 2C). This effect was abolished when iNOS activity was inhibited with LNMMA (Fig. 2C).

We also studied the effect of NO on human VE-cadherin/p120-catenin/β-catenin complex expression and endothelial barrier properties in confluent HUVEC monolayers. Analysis of HUVEC TX-fractions, as well as total cell lysates, was done as described for H5V cells. VE-cadherin levels were markedly reduced in both TX fractions and total cell lysates. p120-catenin levels mirrored VE-cadherin levels in all samples (Fig. S2). β-catenin levels were also reduced but to a lesser magnitude compared to the other AJ proteins analysed (Fig. S2, quantitative densitometry analysis). Co-incubations with LNMMA annulled the effect of NO on the abundance of AJ proteins (Fig. S2). Like for H5V cells, IFNγ/LPS treatment of HUVEC cells induced NO production and increased permeability of the HUVEC cells to a high molecular mass marker (Ig-HRP)(Fig. S2 panel E).

These observations suggest that IFNγ/LPS effect on the levels of β-catenin, p120-catenin and VE-cadherin are attributable to the presence of an active iNOS and the production of high levels of NO (Fig. 2 and S2). In conclusion, the data reported in Fig. 2 and S2 shows that NO regulates expression of AJ proteins and induces changes in the endothelial barrier properties.

### Nitric Oxide Induction Promotes Post-translational Modifications of AJ Proteins

**Figure 3:**
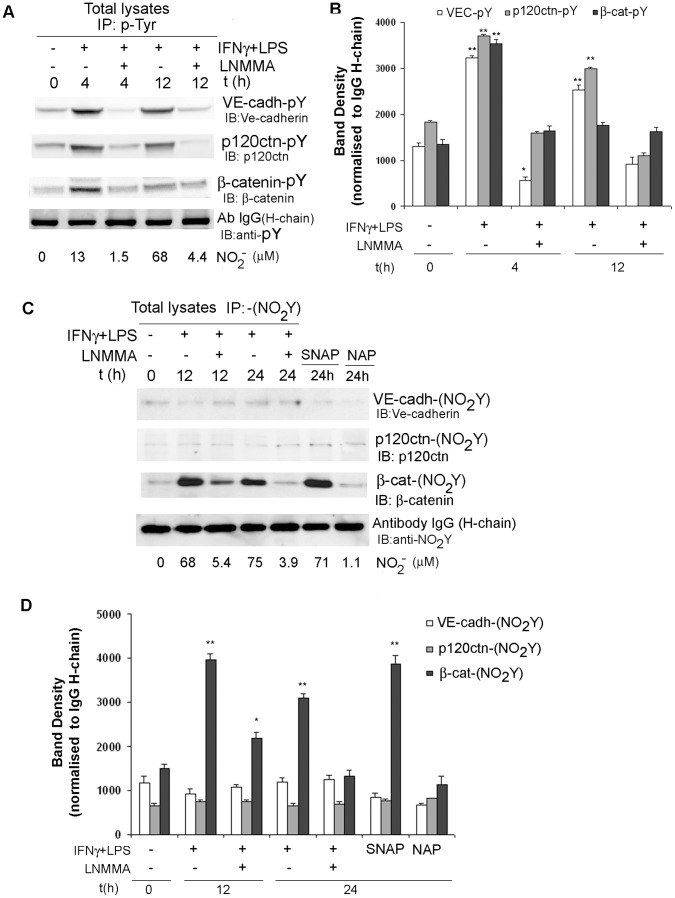
NO induction promotes post-translational modifications of VE-cadherin/catenin proteins. **A.** NO stimulates tyrosine phosphorylation of VE-cadherin/p120-ctn/β-catenin proteins. H5V cells were incubated with IFNγ/LPS, IFNγ/LPS/LNMMA and total cell lysates obtained. Equal amounts of proteins were immunoprecipitated using an antibody that specifically recognises tyrosine phosphorylation Precipitated proteins were analysed by western blot for VE-cadherin, p120-ctn and β-catenin expression using specific antibodies. Antibody (antityr-phosphorylation) IgG levels were used as a loading control. **B.** Graph represents the densitometry analysis of Panel A western blots. Antibody (antityr-phosphorylation) IgG (H-chain) levels were used as a loading control. The significance level was set at P<0.05 (*P<0.05; **P<0.01). **C.** NO promotes β-catenin nitration. H5V cells were incubated with IFNγ/LPS, IFNγ/LPS/LNMMA and total cell lysates obtained. Equal amounts of proteins were taken, and nitrated proteins were immunoprecipitated using an antibody that specifically recognises nitrotyrosine. Precipitated proteins were analysed by western blot for VE-cadherin, p120-ctn and β-catenin expression using specific antibodies. Antibody (antityr-nitration) IgG levels were used as a loading control. **D.** Graph represents the densitometry analysis of Panel C western blots. Antibody (antityr-nitration) IgG (H-chain) levels were used as a loading control. The significance level was set at P<0.05 (*P<0.05; **P<0.01).

Post-translational modifications of AJ proteins are associated with impaired barrier function. For instance, inflammatory mediators such as TGF-β, VEGF, histamine and peroxynitrite increase vascular permeability via AJ phosphorylation or nitration [27]. We, therefore, examine the phosphorylation and nitration state of VE-cadherin, p120-catenin and β-catenin in H5V cells stimulated to produce NO. IFNγ/LPS treatment increased the phosphorylation levels of all AJ proteins. Using quantitative densitometry, tyrosine phosphorylation of VE-cadherin, p120-catenin, and β-catenin, was increased 2.4-, 2.05- and 2.6-fold respectively, in response to IFNγ/LPS treatment for 4 h (Fig. 3A and 3B). Addition of the iNOS inhibitor, LNMMA abolished IFNγ/LPS effect on nitrite production and AJ phosphorylation. These data suggest that increases in AJ phosphorylation can be mediated through activation of iNOS and subsequent NO production (Fig. 3B). However, NO effect on phosphorylation of AJ proteins was transient and reversible. Extended stimulation (12 h) results in a decrease of VE-cadherin (2.0 fold), p120-catenin (1.8 fold) and β-catenin (1.1 fold) phosphorylation levels, in contrast to results obtained with short simulations (Fig. 3A and 3B).

We also determined whether IFNγ/LPS treatment promotes nitration of AJ proteins in H5V cells. Protein tyrosine phosphorylation and nitration might be mutually exclusive. Therefore, we decided to establish protein nitration in cells stimulated for longer periods (12 h–24 h). Tyrosine nitrated proteins were immunoprecipitated from cell lysates with an antibody that specifically recognises nitrotyrosine. The immuprecipitate was then immunoblotted for VE-cadherin, p120-catenin and β-catenin. We failed to detected nitration of either VE-cadherin or p120-catenin in IFNγ/LPS treated cells, or in cells exposed to NO releasing compound SNAP (Fig. 3C and 3D). However, we observed significant increases in β-catenin nitration levels in cells stimulated with IFNγ/LPS (2.6 fold, 12 h) or SNAP (2.5 fold) (Fig. 3C and 3D). Nonetheless, we detected a reduction of nitrated β-catenin levels after 24 h incubation (2.07 fold) with IFNγ/LPS in comparison to the levels detected for 12 h incubations (Fig. 3C and D). Nitration of β-catenin remained at basal levels when NO production or release was prevented by LNMMA or NAP respectively (Fig. 3C and D). These results suggested a direct effect of NO and its derivatives on the post-translational modifications of β-catenin.

### Nitric Oxide Induces Nuclear Accumulation of β-catenin

**Figure 4:**
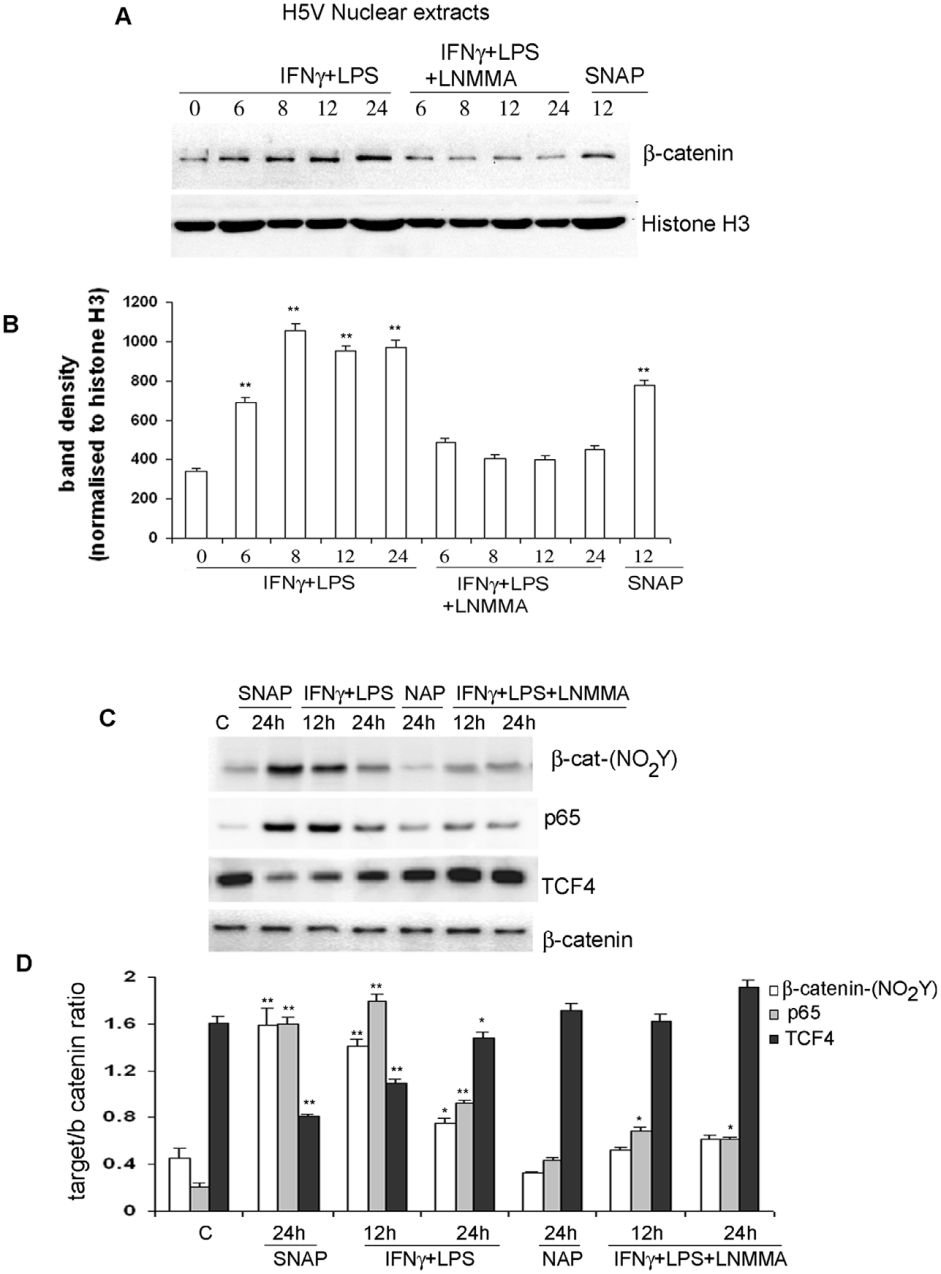
NO influences β-catenin nuclear levels and association to its nuclear partners. **A.** Effect of Nitric oxide on β-catenin nuclear translocation. H5V confluent monolayers were incubated with either IFNγ/LPS or IFNγ/LPS/LNMMA. Cells were harvested at different incubation times and nuclear extracts were obtained as described in Methods. Equals amounts of proteins were analysed by protein blotting for β-catenin and Histone H3 (loading control) levels. **B.** Graph represents the densitometry analysis of Panel A western blots. Histone H3 was used as a loading control. The significance level was set at P<0.05 (*P<0.05; **P<0.01). **C.** Effect of NO on β-catenin nitration and association to its nuclear partners in H5V cells. Confluent H5V cells were incubated with NO donors (SNAP) or IFNγ/LPS combination. The specificity of the iNOS-derived NO effect was assayed by incubating the cells with the NOS inhibitor LNMMA. NO donor specificity was assessed by incubating the cells with the structurally related non-releasing compound NAP. After 24 h incubation, β-catenin was immunoprecipitated from the monolayers and tyrosine nitration and β-catenin levels were detected by immunoblot. The amounts of TCF4 or p65 protein associated to the β-catenin immunocomplexes were assessed by immunoblots. **D.** Graph represents the densitometry analysis of Panel C western blots. β-catenin was used as a loading control. The significance level was set at P<0.05 (*P<0.05; **P<0.01).

β-catenin phosphorylation reduces its affinity for the cadherin cytoplasmic tail and targets the protein either for degradation by the ubiquitin/proteasome pathway (serine/threonine phosphorylation) or for translocation into the nucleus (tyrosine phosphorylation) [15], [28]. In particular, tyrosine phosphorylation of β-catenin (residue Y654) severs cadherin binding and stimulates the association of β-catenin with nuclear transcription factors following translocation to the nucleus [29], [30]. We thus investigate whether β-catenin, once released from VE-cadherin complex, was translocated to the nucleus under NO stimulation. As showed in Fig. 4A, increases in NO levels promoted time-dependent increases in the nuclear translocation of β-catenin (∼ 2.0 to 3.2 fold). NO seems to mediate this effect since nuclear levels of β-catenin were restored when cells were incubated with IFNγ/LPS/LNMMA (Fig. 4B). Nuclear β-catenin increases were also observed when H5V cells were exposed to SNAP for 24 h (2.3 fold), suggesting that NO influences the nuclear location of endothelial β-catenin (Fig. 4B).

### Endogenous Nitration Changes β-catenin Association with its Partners in the Nucleus of IFNγ/LPS -activated Endothelial Cells

Post-translational modification of β-catenin influences its cellular location and interaction with different partners such as TCF4 and NFκB proteins [14], [15]. We, therefore, determined the levels of TCF4 and p65 proteins associated with nitrated β-catenin in H5V cells stimulated to produce NO or exposed to the NO donor SNAP. H5V cell lysates were immunoprecipitated with anti-β-catenin antibody and precipitates analysed for TCF4 and p65 levels using specific antibodies. An anti-nitrotyrosine antibody was used to correlate β-catenin nitration levels to changes in β-catenin association with its nuclear partners.

**Figure 5:**
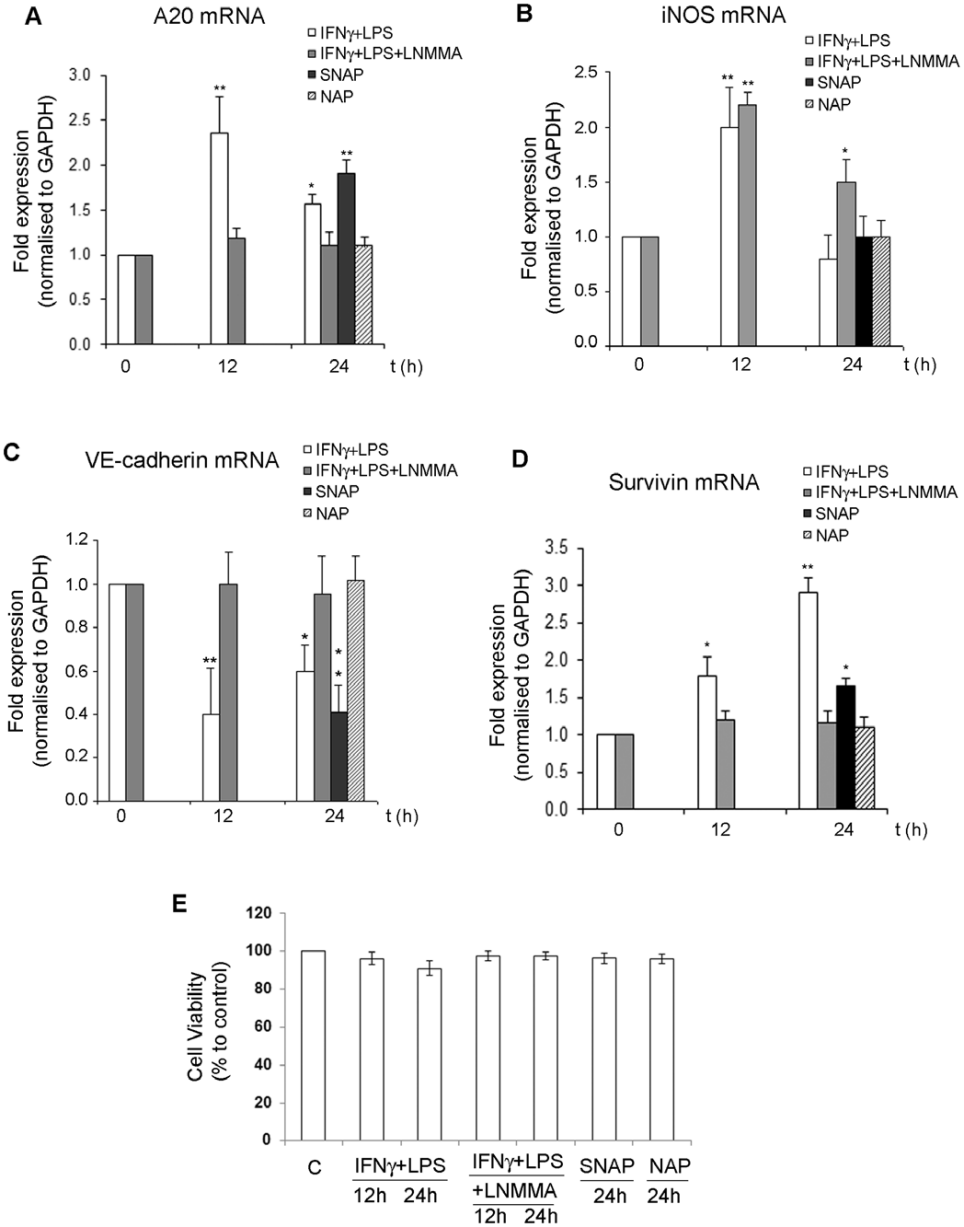
Effect of NO on cell viability and β-catenin mediated gene expression A–D. NO effect on β-catenin mediated gene expression of NFκB and Wnt targets. H5V confluent monolayers were incubated with IFNγ/LPS, IFNγ/LPS/LNMMA, SNAP or NAP for 12 and 24 h. RNA was extracted from the cells and transcript levels of the antiapoptotic molecule A20 (panel A), iNOS (panel B), VE-cadherin (panel C) and survivin (panel D) quantified by RT-PCR. All values are normalised to GAPDH values and expressed as Fold to the 0 h stimulation value. **E.** NO does not affect the viability of H5V cells. H5V cells were treated with IFNγ/LPS or IFNγ/LPS/LNMMA for 12 h or 24 h. Viability was measured by MTT assay. Cell viability is expressed as percentage of viable cells with respect to control cells (100%). Results are means ± s.e.m of three independent experiments. The effect of NO donor SNAP and its structurally related non-releasing compound NAP is also shown.

Nitration seems to influence the abundance of transcription factors associated with endothelial β-catenin (Fig. 4C and D). In unstimulated cells, poorly nitrated β-catenin associates with TCF4 protein. However, we observed that 12h-stimulations with IFNγ/LPS or 24h-incubations with SNAP reduced the levels of TCF4 (1.5 fold and 2.0 fold reduction respectively), and caused an increase in p65 levels (10.4 fold and 11.8 fold increase respectively) associated with β-catenin. These experimental conditions promoted maximal β-catenin nitration (Fig. 5A and 5B). Longer IFNγ/LPS stimulations increased TCF4 levels (1.3 fold) and reduced p65 levels (1.9 fold) associated with β-catenin when compared to shorter incubation periods. Interestingly, the above TCF4 levels did not reach those observed in untreated samples when cells were stimulated for 24 h; probably because β-catenin was still nitrated but to a lower extent that at 12 h (Fig. 5A and 5B). These results suggest that nitration favours association of β-catenin with NFκB proteins, while reducing its association with Wnt pathway transcription factors.

The observed effects seem to be mediated by NO, since both NAP and LNMMA restored the levels of TCF4 protein associated with β-catenin to those observed in unstimulated samples (Fig. 5A and 5B). Nonetheless, we observed that LNMMA incubations did not restore normal levels of p65 protein associated with β-catenin. This can be explained as LNMMA is not an inhibitor of the NFκB pathway; therefore we can assume that p65 is still translocated to the nucleus upon IFNγ/LPS/LNMMA stimulation and binds the basal pool of nitrated β-catenin. This effect is not observed in control samples because the NFκB pathway is inactive in resting cells.

### Activation of iNOS Promotes Transcription of NFκB/β-catenin Pathway Targets without Affecting Endothelial Cell Viability

Given that nitration influences association of β-catenin with NFκB and Wnt pathway transcription factors, we checked whether iNOS activation influences the transcription levels of Wnt and NFκB pathway target genes in endothelial cells.

We selected the antiapoptotic molecule A20 and iNOS, both known targets of the NFκB pathway, to verify NFκB downstream target gene expression in stimulated H5V cells [31], [13]. We observed significant increases in A20 and iNOS transcript levels after 12 h incubation with IFNγ/LPS, a time point that corresponds to maximal β-catenin association to p65 (Fig. 5A and B). The A20 transcript levels were still significantly higher in IFNγ/LPS and SNAP treated cells after 24 h incubations, but of a lesser magnitude when compared to 12 h incubations with IFNγ/LPS (Fig. 5A). Inhibition of NOS activity or NAP incubations did not change the basal levels of the A20 transcript. NO effect in A20 transcription levels parallels the abundance of β-catenin/p65 complexes observed at 12 h and 24 h IFNγ/LPS incubations (Fig. 4C).

In the case of iNOS transcript, its level at 12 h resembles the abundance of β-catenin/p65 complexes; whereas a reduction of its transcription is observed at longer stimulations times. It has been widely described that high levels of NO inhibit NFκB transcriptional activity on the iNOS promoter, a feedback mechanism that protects the cell from sustained iNOS activation [32]. Accordingly, we observed a reduction of iNOS transcript levels after 24 h of NO production that correlates with maximal accumulation of nitrites in the cell media (Fig. 5B). The 12 h inhibition of NOS enzymatic activity did not change the abundance of iNOS transcripts (Fig. 5B). After 24 h incubation with IFNγ/LPS/LNMMA, an increase in iNOS transcript levels is still detected due to the reduction on NO levels by inhibition of iNOS activity (Fig. 5B). Incubation of H5V cells with NAP or SNAP did not change the transcript levels of murine iNOS gene (Fig. 5B).

Since both murine [−1330, −779] and human [−845, −1049, −2760] VE-cadherin promoter region contains several binding sites for TCF4 proteins; we investigated the expression of VE-cadherin as a target for the Wnt pathway [33], [34]. As reported before by us, 24 h SNAP incubations reduced levels of VE-cadherin transcript while NAP has no effect [20], (Fig. 5C). Transcription of murine VE-cadherin was reduced after 12 h incubation with IFNγ/LPS (Fig. 5C). At 24 h IFNγ/LPS stimulation, an increase in VE-cadherin transcript levels is observed compared to 12 h, but not sufficient to reach the levels of untreated cells. Inhibition of NOS activity for 12 h or 24 h did not change the basal levels of murine VE-cadherin (Fig. 5C). In conclusion, the reduction in VE-cadherin transcription correlates with the reduction of β-catenin/TCF4 complexes reported in this study.

Finally, the levels of the antiapoptotic protein Survivin were also measured, as it is a target for both the NFκB and Wnt pathways [35], [36]. As expected, challenging the cells with IFNγ/LPS increased the levels of Survivin transcript (Fig. 5D). Inhibition of NOS activity or incubations with NAP did not change the basal levels of Survivin. Higher levels of Survivin mRNA were observed at 24 h when compared to 12 h IFNγ/LPS stimulations (Fig. 5D). The above suggests that NFκB and Wnt pathways synergistically protect endothelial cells against increased NO production.

Given that expression of genes involved in cell survival and the antiapoptotic response were upregulated in conditions were nitration was favoured, we tested whether iNOS activation influences viability of the cells. We observed that although IFNγ/LPS stimulations slightly reduced the percentage of viable cells, these reductions were not statistically significant. Viability of the cells was not altered by IFNγ/LPS/LNMMA, NAP or SNAP treatments. In conclusion, conditions that favour release of high levels of NO and β-catenin nitration did not affect endothelial cell viability. Overall these results suggest that, in endothelial cells, β-catenin acts as a sensor of nitrating stress favouring the transcription of cytoprotective genes to limit endothelial cell damage.

## Discussion

NO, either derived from endothelium or inflammatory cells, is an important signalling mediator in many endothelial cell (EC) processes [1]–[3]. In EC, β-catenin has a dual role as an adherent protein at the cell-cell junctions stabilizing the VE-cadherin complex and as nuclear co-modulator of the Wnt pathway [11], [14], [15]. Recently, it has also been shown that β-catenin can directly interact with NFκB proteins suggesting a role for β-catenin modulating gene expression during inflammation [12]. Our data demonstrate how iNOS, when activated in macrophages or ECs, induces changes in endothelial permeability associated with post-translational modifications of the β-catenin protein that promoted its nuclear translocation, association with NFκB proteins and regulation of β-catenin-mediated gene transcription. NO, similar to many agents including histamine and VEGF, increases permeability without affecting endothelial-cell viability or functional responses [17], [37].

β-catenin interacts with different transcription factors depending on the functional condition of the cells (such as hypoxia, TGF signalling, Wnt ligands) determining the downstream cellular responses [11]. In inflammation, cytoprotective genes activated by the NFκB pathway, oppose cell death and pro-inflammatory changes induced by cytokines in endothelial cells [1], [2]. In this context, NO regulation of β-catenin transcriptional activity associated with NFκB and Wnt pathways may transduce survival signals, critically maintaining EC viability in inflammatory conditions associated with vascular injury.

Endothelial cell survival is also required during vascular remodelling and angiogenesis [38]. NO may promote tumour-associated angiogenesis by increasing vascular permeability and expression of cytoprotective genes such as Survivin and the zinc finger molecule A20. Interestingly, a positive correlation between NO production and tumour angiogenesis has been reported in wide range of human cancers [39]. It has been reported that NO activates the transcription factor hypoxia-inducible factor 1 α (HIF1 α), which, in turn, upregulates vascular endothelial growth factor (VEGF) gene expression [39]. In endothelial cells, VEGF increases AJ tyrosine phosphorylation, vascular permeability and endothelial cell migration [40]. VEGF regulation of AJs is considered to be important for the establishment of angiogenic activated ECs. These suggest that high levels of NO regardless of its cellular origin may lead to activation of a pathway that promotes angiogenesis. Accordingly, recent reports have demonstrated that tyrosine phosphorylation of VE-cadherin is key to switching of EC phenotype from adhesive to motile [41].

Integrity of the endothelium and control of its barrier properties is critical for modulating the inflammatory response [27]. Adherent junctions (AJs) contribute to the semipermeable characteristic of the endothelium. Clusters of surface VE-cadherin promote the formation of AJs. They constitute multimolecular complexes that comprise signalling, regulatory and scaffolding proteins including p120-catenin and β-catenin [27]. It is generally accepted that the tyrosine phosphorylation of VE-cadherin, leads to its detachment from the actin cytoskeleton, to an increase in paracellular permeability and ultimately to the weakening of AJs [42]. In ECs exposed to high levels of NO, we observed a reduction in the levels of VE-cadherin, p120-catenin and β-catenin in the TX-insoluble fractions that represents the proteins tightly associated with the cell cytoskeleton, as well as in TX-soluble fractions and total cell lysates. These events were associated to tyrosine phosphorylation of VE-cadherin and disruption of endothelial barrier properties. Other permeability-increasing agents such as histamine, tumour necrosis factor-α (TNFα), platelet-activating factor (PAF) and VEGF also induce tyrosine phosphorylation of the VE-cadherin/catenin complex and AJ opening [37], [40], [42]. Similar to the above permeability inducing agents, NO also promotes β-catenin and p120-catenin tyrosine phosphorylation.

In several systems, the tyrosine phosphorylation of β-catenin reduces its affinity for cadherin’s cytoplasmic tail [15], [28]. At present, β-catenin binding to VE-cadherin is known to be essential for the control of barrier properties of the endothelium [42]. It is possible that NO effects on β-catenin post-translational modifications compromises cytoskeletal organization and impairs the barrier function of the endothelium.

Recruitment of p120-catenin to AJs promotes cadherin clustering and strengthens adhesion [43]. Treatment of endothelial monolayers with inflammatory mediators has been demonstrated to increase endothelial permeability, which has been correlated with changes in p120-catenin phosphorylation [44]. Furthermore, tyrosine-phosphorylation defective p120-catenin mutants efficiently bind and stabilize E-cadherin at the cell surface of normal cells [45]. We observed that NO induced tyrosine phosphorylation of p120-catenin and decreased the levels of VE-cadherin and p120-catenin proteins. In ECs, p120-catenin levels function as a set point for cadherin expression levels, by preventing cadherin degradation via an endosomal–lysosomal pathway [46]. In turn, cadherins antagonize β-catenin signalling by sequestering β-catenin to the cell-cell junctions [10], [42]. Therefore NO regulation of p120-catenin levels reported here, may serve as a mechanisms to control β-catenin availability to inflammatory signal transduction pathways.

Tyrosine phosphorylation of AJs in response to different stimuli is mediated by several tyrosine kinases including Src, PYK2, PAK and RAC kinases [15], [41], [42], [44]. Presently, a direct effect of iNOS activation on the activity of these kinases has not been proved. However, NO enhances EGF receptor-and cGMP/protein kinase G (PKG) -dependent protein tyrosine phosphorylation of substrates [4]. Further studies are needed to confirm whether NO phosphorylation of AJ proteins is mediated by EGFR or cGMP/PKG signalling pathways.

Nitration of proteins has been described in inflammatory pathologies characterized by high levels of NO [2], [47]. We demonstrated that nitration of endothelial β-catenin influenced its nuclear association with transcription factors of the TCF and NFκB pathways. In our experiments, tyrosine nitration of β-catenin promoted its recruitment to p65 transcription complexes. This effect was concomitant with increases in iNOS transcript and a significant reduction in VE-cadherin transcript.

Loss of VE-cadherin at cell-cell contacts, vascular injury and increase in nitrotyrosine formation are among the detrimental effects caused by oxidative stress [3], [27], [42], [47]. In cancer cells an inhibitory effect of β-catenin in the transcriptional activity of NFκB at the iNOS promoter has been described [48]. Nitration of β-catenin may have a protecting effect on the endothelium favouring p65/β-catenin interaction and limiting the magnitude of iNOS induction and reactive oxygen species formation. Accordingly, we observed accumulation of β-catenin/TCF4 complexes and increased expression of Wnt downstream targets associated with a decreased in β-catenin nitration levels.

Exposure of cells to NO increases the production of superoxide anion and consequently of the pool of nitrogen dioxide radical, involve in tyrosine nitration of proteins [47]. This provides a mechanism by which NO provokes tyrosine nitration of β-catenin when the pool of NO and subsequently nitrogen dioxide radical predominates.

β-catenin/TCF signalling promotes vascular remodelling after injury and survival of ECs [49]. Previous studies described that β-catenin/TCF4 complexes are more abundant in the nucleus of VE-cadherin-null cells than in VE-cadherin-positive cells [50]. Consistent with these reports, our data shows increased formation of TCF/β-catenin complexes associated with a reduction in VE-cadherin protein expression and high levels of NO.

Human and murine endothelial cells have a functional Wnt signalling pathway that could play a role in growth and differentiation of normal endothelium as well as pathological angiogenesis [51]. Thus, in analogy to Wnt proteins, NO may regulate angiogenesis and permeability by controlling nuclear translocation of β-catenin and its association to different transcription factors. Induction of Survivin and the antiapoptotic molecule A20 by the NO β-catenin axis may provide a cytoprotective mechanism during tumour angiogenesis. Molecular antagonism of the NO-β-catenin pathway may sensitise tumour cells to therapy induced apoptosis.

In conclusion, we demonstrated that iNOS-derived NO regulates endothelial β-catenin functions. Our data suggest that a NO signalling pathway exist in ECs that recognizes the nitrating stress. β-catenin acts as sensor protein transmitting NO signal by switching its role from adherent protein to nuclear transcription factor, regulating gene expression. This mechanism might support EC survival during inflammation limiting cell injury associated with high levels of NO. Conversely in cancer cells, NO regulation of β-catenin may provide a mechanism of cytoprotection to maintain a pool of ECs necessary to promote tumour growth. This study may help to increase the understanding of the pluripotential roles of NO in vascular physiology and pathophysiology.

## Materials and Methods

### Cells and Reagents

H5V, a murine microvascular endothelial cell line [52], was cultured, as described before [20], in Dulbecco’s modified Eagle's medium (DMEM) with 10% foetal calf serum (FCS), glutamine 2 mM, penicillin (100 U/ml) and streptomycin (100 mg/ml) at 37°C in a 5% CO_2_ atmosphere. Human umbilical vein endothelial cells (HUVEC) [77, ATCC Number: CRL-1730], were cultured as described before [53], in M199 and 20% newborn calf serum, endothelial cell growth supplement, and heparin on gelatin-coated tissue culture vessels. The macrophage RAW 264.7 cell line (ATCC Number: TIB-71), was cultured in high glucose Dulbecco’s modified Eagle’s medium supplemented with 5% foetal bovine serum at 37°C in a 5% CO_2_ atmosphere.

Murine recombinant interferon gamma (IFNγ), *Escherichia coli* lipopolisacaride (LPS, serotype 0111: B4), L-N-monomethyl arginine citrate (LNMMA), S-nitroso-N-acetylpenicillamine (SNAP) and N-acetylpenicillamine (NAP) were from Sigma (UK).

### Antibodies

Mouse monoclonal antibody (mAb) to β-catenin (clone 14), mouse mAb to NOS II (clone 6) and mouse mAb to phosphotyrosine residues (clone PY69) were from Transduction Laboratories (USA). Rabbit polyclonal anti-p120 catenin (H-90) was from Santa Cruz Biotechnology (Santa Cruz, CA). Rat mAb to murine VE-cadherin (clone BV13) and mouse mAb to PECAM-1 (clone 7.46) were from Abcam (UK). Mouse anti-TCF4 (clone 6H5-3) and rabbit anti-p65 antibodies were from Millipore (UK). IgG-HRP peroxidase conjugates were from Amersham (UK). Rabbit polyclonal anti-nitrotyrosine antibody was from Cayman Chemical (Ann arbour, MI) and rabbit polyclonal Histone (H3) antibody and rabbit mAb to α-tubulin (11H10) were from Cell Signaling Technology (UK).

### Nitrite Measurement

The nitrite content in cell culture supernates was determined using the Griess reagent containing 0.5% sulfanilamide and 0.05% N-(1-naphthyl) ethylenediamine hydrochloride in 45% acetic acid. Nitrite reacts with Griess colour reagent to give a red-violet diazo-dye which is measured spectrophotometrically at 543 nm and nitrite concentration is calculated from a sodium nitrite standard curve. Briefly, 500 µl of cell culture media were taken and added to a plastic cuvette containing 500 µl of Griess reagent and the absorbance was measured at 543 nm [54].

### Viability Assay

For viability assays, H5V cells (15×10^3^ cells/well) were seeded in triplicate into 96-well plates. On the following day, cells were incubated with IFNγ/LPS, IFNγ/LPS/LNMMA, SNAP or NAP. At various time points (0, 12 h, 24 h) viable cells were estimated by 3-(4,5-Dimethylthiazol-2-yl)-2,5-diphenyltetrazolium bromide (MTT) assay. Briefly, 20 µl of MTT (5 mg/ml, Sigma) solution was added to each well (200 µl final volume) 4 hours before the end of the experiment. The medium was then aspirated and the formazan crystals were solubilized with dimethylsulfoxide. Absorbance at 630 nm was subtracted (substrate background) from absorbance at 570 nm in each well. Values of viability of treated cells were expressed as a percentage of that from corresponding control cells (assumed to be 100%).

### Preparation of Protein Samples

Triton X-100 (TX) soluble and insoluble fractions, as well as total cell extracts, were obtained from long confluent endothelial cells as described previously [53]. Briefly, long confluent monolayers were washed twice with Ca^2+^- and Mg^2+^-containing PBS. Cells were extracted for 20 min on ice in lysis buffer containing 40 mM Tris (pH 7.6), 500 mM NaCl, 2 mM CaCl_2_, 1% Nonidet P-40, 1% Triton X-100, 2 mM Na_3_VO_4_, 1 mM PMSF, 20 U/ml aprotinin and 15 µg/ml leupeptin. Cell extracts were then centrifuged at 14000 g for 5 min (4°C) and the supernatant was defined as the TX soluble fraction while the pellets (TX insoluble fraction) were further solubilised in lysis buffer supplemented with 0.02% SDS. For total cell lysates, monolayers were extracted in lysis buffer containing 0.5% SDS. For experiments in which tyrosine phosphorylation was studied, cells were then washed twice in Ca^2+^- and Mg^2+^-containing PBS and disrupted on ice for 20 min with lysis buffer. Cell extracts were then centrifuged at 14000 g for 5 min (4°C) and used in immunoprecipitation assays.

Nuclear proteins were isolated using NE-PER nuclear and cytoplasmic extraction reagents according to the manufacturer protocol (Pierce Biotech).

### Immunoprecipitation of Proteins

Immunoprecipitation was performed as described previously [20], with some modifications. Briefly, equal amounts of protein samples (total cell lysates or nuclear extracts) were precleared on protein A-Sepharose for 1 h at room temperature. The supernatant, separated by brief centrifugation at 10 000 r.p.m, was incubated with protein A-Sepharose coupled to mAbs for 3 h at 4°C on a rocking platform. Bead-protein complexes were then washed 3 times in TBS containing protease inhibitors. Bead-immunocomplexes were collected by brief centrifugation at 10 000 r.p.m, resuspended in Laemmli sample buffer containing 2-mercaptoethanol (5% final concentration) and boiled for 5 min.

### SDS-PAGE Electrophoresis and Western Blot

For immunoblotting 10 µg of protein samples diluted in Laemmli buffer (total cell lysates, immunocomplexes or nuclear cell extracts) were electrophoresed on a 7.5% SDS-polyacrylamide gel. The gel was then incubated for 30 min (2×15 min) in transfer buffer containing 1 mM CaCl_2_. Separated proteins were electroblotted onto a PVF membrane. Membranes were incubated overnight with blocking buffer (5% BSA dilute in 1 mM Ca^+2^- and Mg^+2^-containing 0.1% Tween-20-TBS (CaMgTTBS) and subsequently incubated for 1 hour at room temperature with the appropriate primary antibody diluted in blocking buffer. Blots were then incubated for 1 h at room temperature with the correspondent IgG horseradish peroxidase secondary antibody diluted in blocking buffer. Between incubation steps, membranes were washed several times with CaMgTTBS buffer. Blots were analysed for α-tubulin or H3 (Cell Signaling Technology) to normalize the protein load in each well. Immunoreactive bands were visualized by ECL using a Chemidoc System Bio-Rad Imager and quantified by Quantity One Imaging software (Bio-Rad, UK) as a function of volume data (intensity/mm^2^). The Volume Rectangle Tool was used to measure the total signal intensity inside a boundary drawn around the bands without overlapping adjacent bands. Background was subtracted from each band volume by using local background subtraction. Intensities of bands acquired from each protein extract were normalised against corresponding values for bands of the house-keeping protein. Results were expressed as Band Density normalised to a house-keeping protein and are expressed as Intensity per square millimetres (INT*mm^2^).

### In vitro Permeability Analysis

To analyse the effect of iNOS activation on cellular permeability, H5V cells (3×10^4^ cell/cm^2^ at seeding) were cultured for 6 days in Transwell units (with polycarbonate filters, 0.4 µm pores; Costar) [20], [53]. At the start of the experiment, the culture medium in the lower and upper compartment was replaced with medium containing the stimulating compounds. TNFα (100 ng/mL) was used as positive control. After 23 h incubation, HRP conjugated to goat immunoglobulin (8 µg/ml initial concentration in the upper chamber; MW = 200 kDa; specific activity 28 units/ml) was added to the upper compartment. After 1 h further incubation at 37°C, the medium in the lower compartment was assayed for the presence of HRP activity using OPD as chromogenic substrate.

### RNA Isolation and RT-PCR

Dnase-I treated RNA was reverse transcribed into cDNA before assessing VE-cadherin, antiapoptotic molecule A20, survivin and iNOS expression using specific primer pairs (Beacon Design 2.0, Premier Biosoft). RT-PCR amplification was performed in triplicate in 96-well plates in a BioRad IQ iCycler. Serial dilutions of cDNA were used to plot a calibration curve, and gene expression quantified by plotting threshold cycle values. Expression levels were normalized to values obtained for the housekeeping gene (GAPDH*)*.

### Statistical Analysis

Data distribution was assessed for normality using the Ryan Joiner and Kolmogorov Smirnov tests. Data are reported as means ± standard errors of the mean (SEM). For normally distributed data, a t-test was used to determine the significance of differences between groups. All data analysis was performed using SPSS version 10.0 (SPSS Chicago IL). Differences were considered significant at the P≤0.05 levels.

## Supporting Information

Figure S1 **Densitometric analysis of immunoblots shown in** **Figure 2** **.** Protein bands were visualized using a ChemiDoc System Bio-Rad Imager (Bio-Rad) and quantified by Quantity One® Imaging software (Bio-Rad) as described in Methods. Results were expressed as Band Density normalised to α-tubulin and are expressed as Intensity per square millimetres (INT*mm^2^). Statistical analysis was done using a t-test. The significance level was set at P<0.05 (*P<0.05; **P<0.01). **A.** Quantification of iNOS induction in H5V cells stimulated with IFNγ/LPS (Immunoblot image is shown in Fig. 2A) **B.** Quantification of VE-cadherin, p120-catenin and β-catenin levels in the TX-100 soluble fraction of H5V cells. (Immunoblot image is shown in Fig. 2B) **C.** Quantification of VE-cadherin, p120-catenin and β-catenin levels in the TX-100 insoluble fraction of H5V cells. (Immunoblot image is shown in Fig. 2B) **D.** Quantification of VE-cadherin, p120-catenin and β-catenin total levels in H5V cells. (Immunoblot image is shown in Fig. 2B)
(TIF)

Figure S2 **NO regulates the expression and function of VE-cadherin/p120catenin/β-catenin complex in H5V cells.** Panel A. Expression of VE-cadherin, p120-catenin (p120-ctn) and β-catenin is reduced in HUVEC cells incubated with IFNγ/LPS. NOS inhibitor LNMMA attenuates NO effect on VE-cadherin/catenin complex. HUVEC TX fractions and total cell lysates were analysed by western blot for VE-cadherin, p120-catenin (p120-cnt) and β-catenin levels using specific antibodies. α-tubulin levels were used as a loading control. Nitrite production was measured using the Griess method and nitrite concentrations expressed in µM. **B–D.** Graphs represent the densitometry analysis of Panel A western blots. α-tubulin was used as a loading control. Protein bands were visualized using a ChemiDoc System Bio-Rad Imager (Bio-Rad) and quantified by Quantity One® Imaging software (Bio-Rad) as described in Methods. Results were expressed as Band Density normalised to α-tubulin and are expressed as Intensity per square millimetres (INT*mm^2^). Statistical analysis was done using a t-test. The significance level was set at P<0.05 (*P<0.05; **P<0.01). **E.** NO stimulates paracellular permeability to IgG-HRP in HUVEC cells. HUVEC cells were grown to confluence in Transwell units and stimulated to produce NO. Monolayer permeability to a tracer (IgG-HRP, 200 KDa) was measured as described in Methods. Control cells were incubated with LNMMA, to inhibit iNOS activation. Statistical analysis was done using a t-test. The significance level was set at P<0.05 (*P<0.05; **P<0.01).
(TIF)
