# Supplementary figures and images for: iNOS Activation Regulates β-catenin Association with Its Partners in Endothelial Cells
Source: PLoS One. 2012 Dec 28;7(12):e52964. doi: 10.1371/journal.pone.0052964 (PMC3532412; doi:10.1371/journal.pone.0052964)

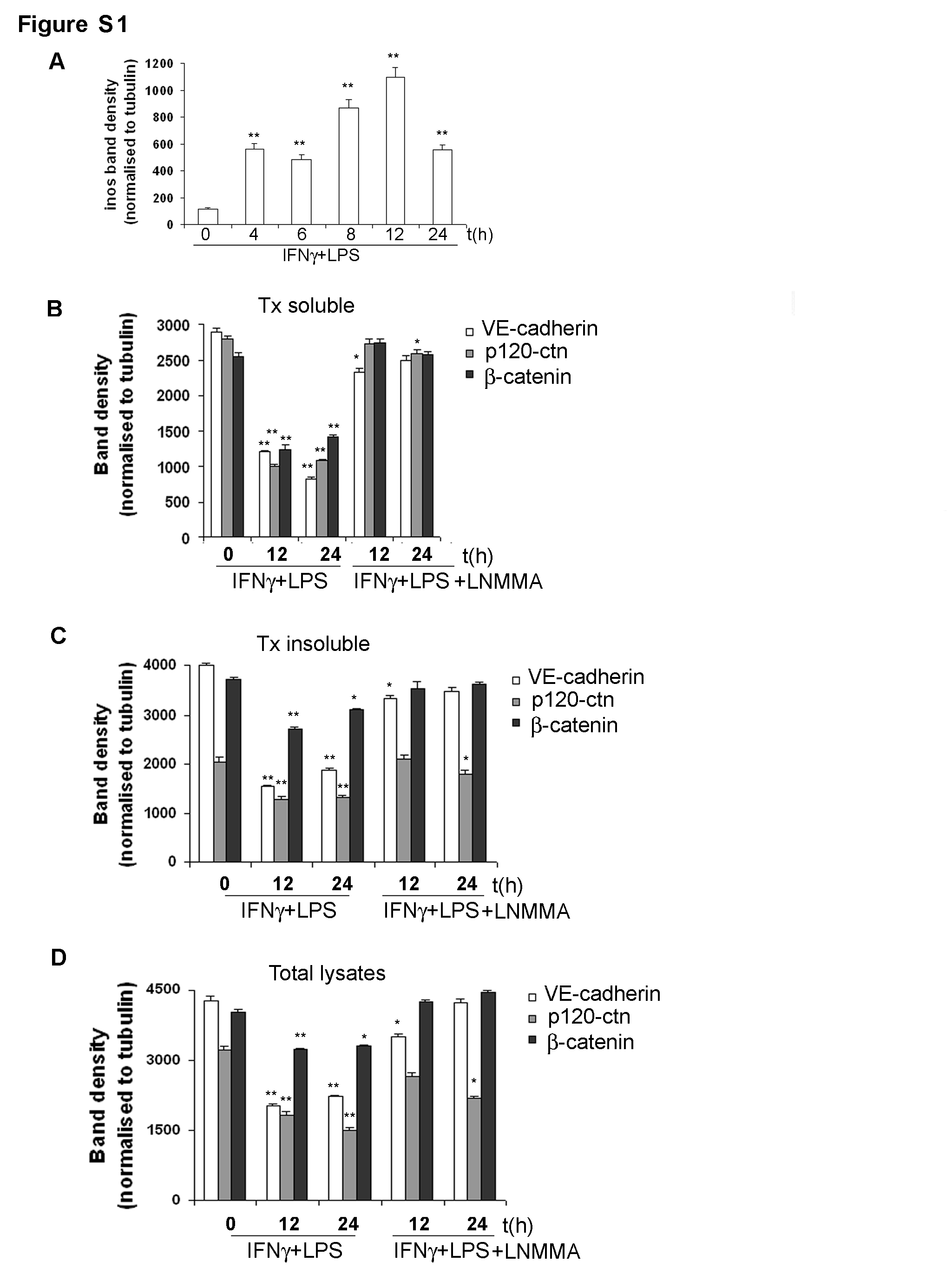

Supplement: Figure S1 — Densitometric analysis of immunoblots shown in Figure 2 . Protein bands were visualized using a ChemiDoc System Bio-Rad Imager (Bio-Rad) and quantified by Quantity One® Imaging software (Bio-Rad) as described in Methods. Results were expressed as Band Density normalised to α-tubulin and are expressed as Intensity per square millimetres (INT*mm2). Statistical analysis was done using a t-test. The significance level was set at P<0.05 (*P<0.05; **P<0.01). A. Quantification of iNOS induction in H5V cells stimulated with IFNγ/LPS (Immunoblot image is shown in Fig. 2A) B. Quantification of VE-cadherin, p120-catenin and β-catenin levels in the TX-100 soluble fraction of H5V cells. (Immunoblot image is shown in Fig. 2B) C. Quantification of VE-cadherin, p120-catenin and β-catenin levels in the TX-100 insoluble fraction of H5V cells. (Immunoblot image is shown in Fig. 2B) D. Quantification of VE-cadherin, p120-catenin and β-catenin total levels in H5V cells. (Immunoblot image is shown in Fig. 2B) (TIF) [file pone.0052964.s001.tif]

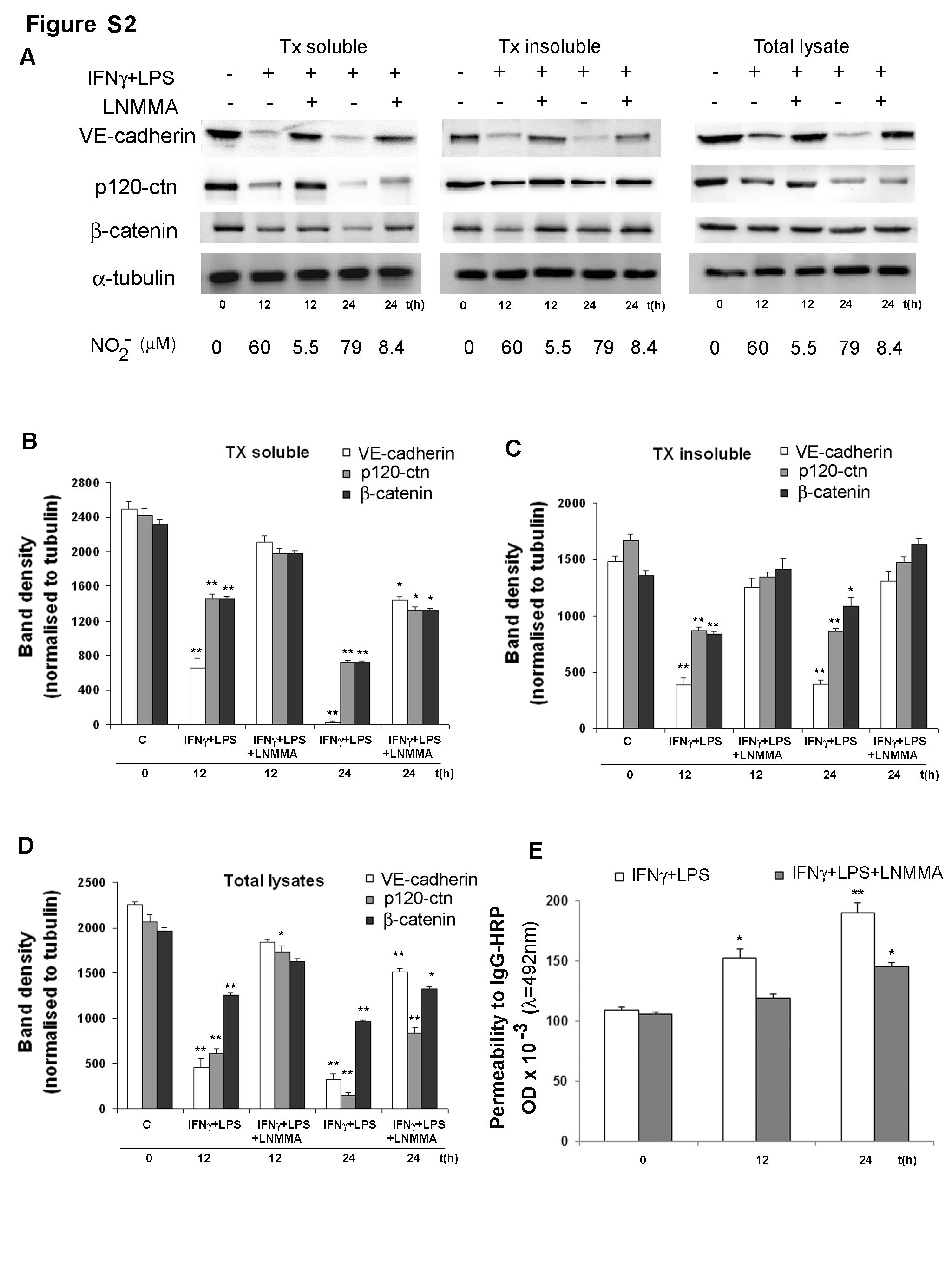

Supplement: Figure S2 — NO regulates the expression and function of VE-cadherin/p120catenin/β-catenin complex in H5V cells. Panel A. Expression of VE-cadherin, p120-catenin (p120-ctn) and β-catenin is reduced in HUVEC cells incubated with IFNγ/LPS. NOS inhibitor LNMMA attenuates NO effect on VE-cadherin/catenin complex. HUVEC TX fractions and total cell lysates were analysed by western blot for VE-cadherin, p120-catenin (p120-cnt) and β-catenin levels using specific antibodies. α-tubulin levels were used as a loading control. Nitrite production was measured using the Griess method and nitrite concentrations expressed in µM. B–D. Graphs represent the densitometry analysis of Panel A western blots. α-tubulin was used as a loading control. Protein bands were visualized using a ChemiDoc System Bio-Rad Imager (Bio-Rad) and quantified by Quantity One® Imaging software (Bio-Rad) as described in Methods. Results were expressed as Band Density normalised to α-tubulin and are expressed as Intensity per square millimetres (INT*mm2). Statistical analysis was done using a t-test. The significance level was set at P<0.05 (*P<0.05; **P<0.01). E. NO stimulates paracellular permeability to IgG-HRP in HUVEC cells. HUVEC cells were grown to confluence in Transwell units and stimulated to produce NO. Monolayer permeability to a tracer (IgG-HRP, 200 KDa) was measured as described in Methods. Control cells were incubated with LNMMA, to inhibit iNOS activation. Statistical analysis was done using a t-test. The significance level was set at P<0.05 (*P<0.05; **P<0.01). (TIF) [file pone.0052964.s002.tif]
